# Power-spectra and cross-frequency coupling changes in visual and Audio-visual acquired equivalence learning

**DOI:** 10.1038/s41598-019-45978-3

**Published:** 2019-07-01

**Authors:** András Puszta, Ákos Pertich, Xénia Katona, Balázs Bodosi, Diána Nyujtó, Zsófia Giricz, Gabriella Eördegh, Attila Nagy

**Affiliations:** 1 Department of Physiology, Faculty of Medicine, University of Szeged, Dóm tér 10, Szeged, Hungary; 2 Department of Oral Biology and Experimental Dental Research, Faculty of Dentistry, University of Szeged, Tisza Lajos krt. 64, Szeged, Hungary

**Keywords:** Perception, Working memory

## Abstract

The three phases of the applied acquired equivalence learning test, i.e. acquisition, retrieval and generalization, investigate the capabilities of humans in associative learning, working memory load and rule-transfer, respectively. Earlier findings denoted the role of different subcortical structures and cortical regions in the visual test. However, there is a lack of information about how multimodal cues modify the EEG-patterns during acquired equivalence learning. To test this we have recorded EEG from 18 healthy volunteers and analyzed the power spectra and the strength of cross-frequency coupling, comparing a unimodal visual-guided and a bimodal, audio-visual-guided paradigm. We found that the changes in the power of the different frequency band oscillations were more critical during the visual paradigm and they showed less synchronized activation compared to the audio-visual paradigm. These findings indicate that multimodal cues require less prominent, but more synchronized cortical contribution, which might be a possible biomarker of forming multimodal associations.

## Introduction

Associative learning is a complex task in which rule-transfer is increased between two superficially dissimilar stimuli (or antecedents) that have previously been associated with similar outcomes (or consequents)^1^. Catherine E. Myers and co-workers developed a learning paradigm (Rutgers Acquired Equivalence Test, also known as the fish-face paradigm), which can be applied to investigate a specific kind of associative learning, visually-guided equivalence learning^2^. This complex test consists of three phases which can be interpreted as better-known tests: The initial phase can be described as an associative learning or trial-and-error learning or rule-based learning task, which primarily requires an intact basal ganglia-network^2,3^. The second part is the retrieval phase, which can be interpreted as working memory maintenance, and the third part is the generalization or rule-transfer phase, which primarily requires an intact hippocampal-mediotemporal system^4,5^. Our research group adapted and modified the Rutgers Acquired Equivalence Test to make it more sensitive, and recently investigated the development of these learning functions in healthy humans and how migraines affect them^6^. This learning paradigm critically requires the normal function of subcortical structures, i.e. hippocampi and basal ganglia^2,7^. Cortical contribution is also necessary in the visually-guided learning paradigm as stimulus representations and associations are stored and (re)activated in stimulus-relevant cortical areas^8–11^. Thus, associative learning requires cooperation between the learning circuit and other task-specific brain areas.

Different areas of the brain must interact to provide the basis for the integration of sensory information, sensory-motor coordination and many other functions that are critical for learning, memory, and perception. Hebb suggested that this is accomplished by forming assemblies of cells whose synaptic linkages are strengthened whenever the cells are activated synchronously^12^. Neuronal oscillations are a natural consequence of forming such cell assemblies via the summation of hundreds of EPSPs and IPSPs, and the cerebral cortex generates multitudes of oscillations at different frequencies mainly through inhibiting spike-trains at a specific frequency. To investigate these oscillations during associative learning, our research group used electroencephalography (EEG), which is a well-known non-invasive monitoring method for investigating the electrical signals generated by large assemblies of neurons and their connections.

A number of investigations have described the EEG-features of the different phases of associative learning and memory. One key feature of the reward-related learning is that the positive feedback elicits beta power increment, while negative feedback causes power increment in both theta and beta power^13,14^. Furthermore, studies in associative learning tasks revealed gamma coherence over parietooccipital areas^15,16^. In working memory tasks, frontal midline theta power increment is a well-known phenomenon^17–19^. Theta/alpha-gamma cross frequency coupling in working memory load was described earlier^20,21^. The tasks described above mainly used unimodal stimulus-pairs to associate, and there is less information about how these patterns change if we apply multimodal stimulus-pairs.

It is well known from earlier studies that both brain structures fundamentally involved in visual associative learning, the basal ganglia and the hippocampi, receive not only visual but also multisensory information^22–25^. A bimodal or multimodal stimulus could be more informative in its complexity than a unimodal stimulus from the environment. The studies referenced above investigated visually-guided equivalence learning and to our knowledge no study has addressed the cortical contribution to multisensory-guided acquired equivalence learning.

Multimodal information could be more informative, than a unimodal stimulus from the environment^26,27^. Multisensory integration occurs at different levels of brain functions. It can be observed at the cellular level^28–31^ in several brain regions, such as the superior colliculus^32^, the basal ganglia^33,34^ the cortex^35^, and the hippocampus^36^, and it can also be observed on the behavioral level^37,38^. In the present study we investigate the effect of multisensory stimuli on associative learning and we ask whether there are any specific changes in the power spectra and in the cross-frequency coupling of the human neocortex in a multisensory task compared to a unimodal visual one^39^.

## Results

Altogether 23 healthy volunteers participated in the investigation. For the biomathematical analysis (including the psychophysical results, time-frequency (TF) results, cross-frequency coupling results, and power - /synchronization index (SI) - performance correlation), the raw electrophysiological data of 18 volunteers were analyzed, as in the other recordings the signal to noise ratio was low, and neither the excessive attempt to clean the data from muscular and ocular artefacts with preprocessing methods described earlier could make them acceptable.

### Data visualization

The electrophysiological results in four different frequency bands (theta (4–7 Hz), alpha (8–13 Hz), beta 14–30 Hz), and gamma (31–70 Hz) will be presented below for each phase (Acquisition, Retrieval and Generalization) of the two (visual and Audio-visual) paradigms.

**Figure 1:**
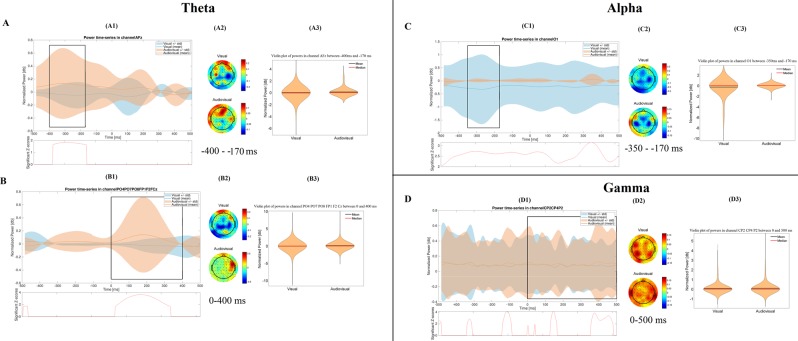
Time-frequency results in the acquisition phase. The figure shows the most important differences, which were found between the visual and the audiovisual tasks. Part (**A**) and Part (**B**) represent the results in the theta band, before and after the given answer, respectively. The (**C**,**D**) parts show the results in the alpha and gamma bands, respectively. Within each part of the figure, there are three different subplots. The subplots with number 1 (A1,B1,C1,D1) show the normalized power-fluctuation of the given channels, and the significant Z-scores between the two time-series calculated with random permutation test before and after 500 ms of the given answer. 0 ms denotes the time point when the answer was given. The subplots with number 2 (A2,B2,C2,D2) show the topographical representation of the mean normalized power in certain time-windows. The red colour indicates power-increase, while the blue one indicates power- decrease compared to baseline-activity. The subplots with 3 (A3,B3,C3,D3) show the violin plot of the normalized powers in the selected time-window and in the selected channels.

**Figure 2:**
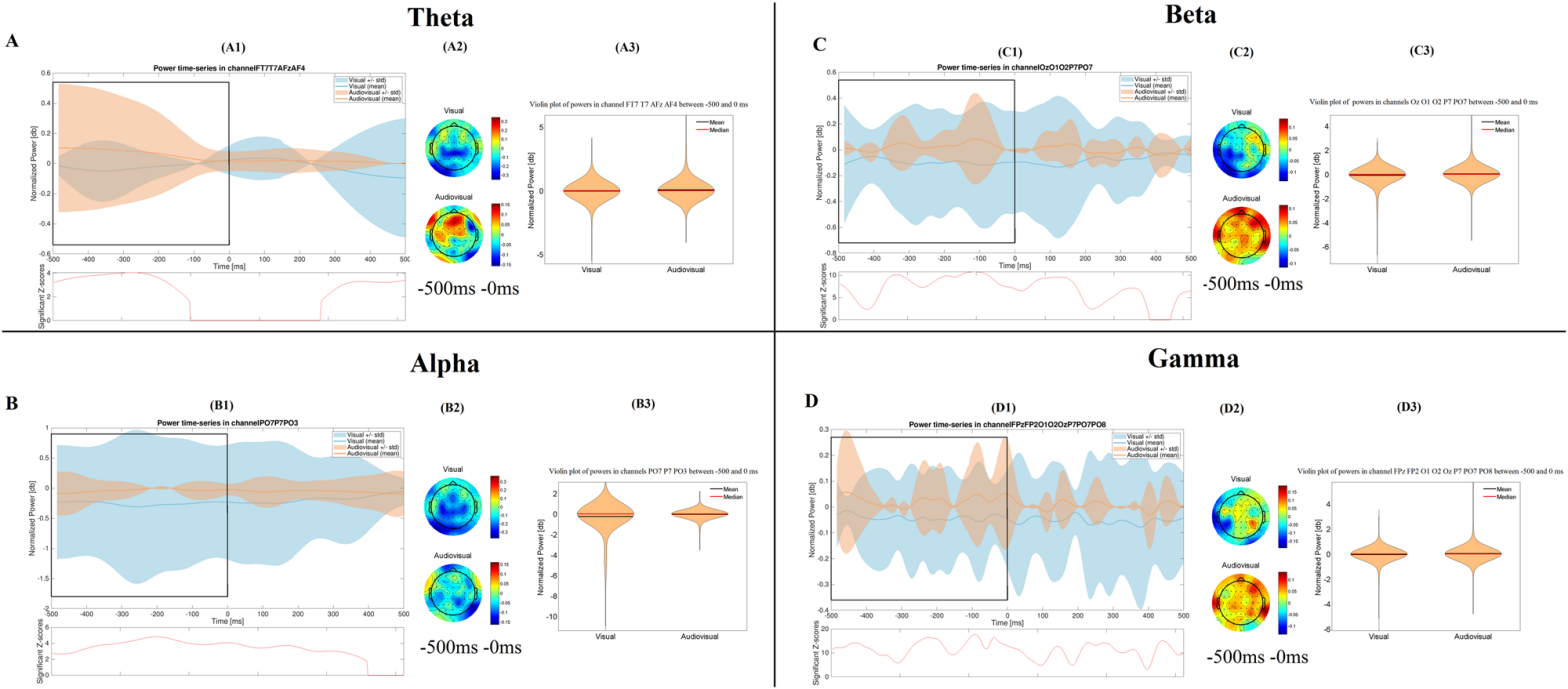
Time-frequency results in the retrieval phase. The figure shows the most important differences, which were found between the visual and the audiovisual task. The (**A**–**D**) parts show the results in the theta, alpha, beta and gamma bands, respectively. Other conventions are same as on Fig. 1.

**Figure 3:**
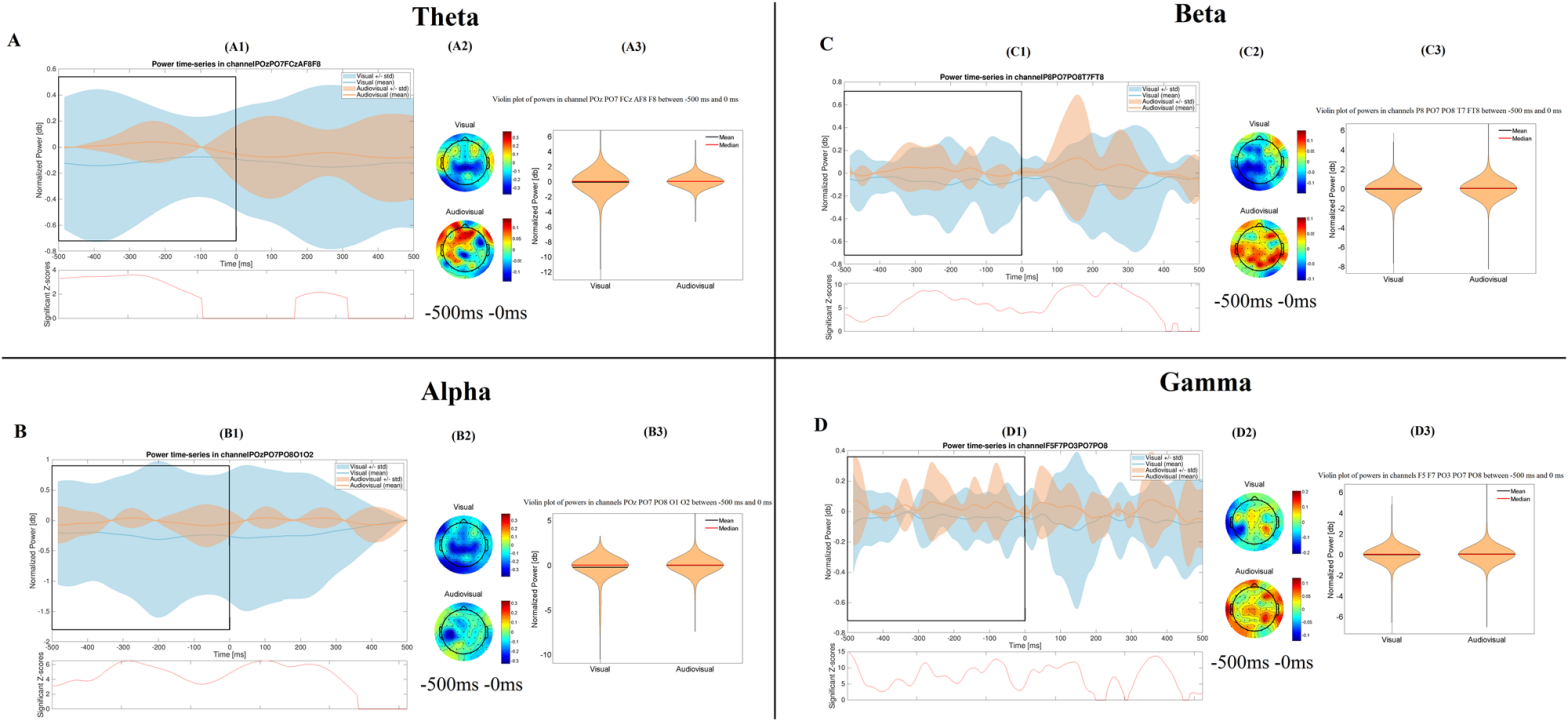
Time-frequency results in the generalization phase. The figure shows the most important differences, which were found between the visual and the audiovisual task. The (**A**–**D**) parts show the results in the theta, alpha, beta and gamma bands, respectively. Other conventions are same as on Fig. 1.

In the time-frequency results, the group-level statistical differences between the time-frequency power spectra of the visual and audiovisual paradigm are presented in each frequency band, and in each phase of the paradigm (See Figs 1–3). In every case, the results are discussed in a range −500 ms–500 ms in case of the Acquisition phase, and −500 ms-0 ms in case of the Retrieval and Generalization phases, where the 0 ms denotes the time of the answer. We will give a detailed description of the statistical differences between the visual and the audiovisual paradigm only in those cases, where we found significant difference between the visual and the audiovisual paradigms. Detailed descriptions of the changes in the time-frequency power spectra in each phase of the visual and Audio-visual paradigm compared to baseline activity are presented in the Supplementary Data (Supplementary Data 1). Furthermore, because of the huge amount of data, our detailed results of the time-frequency analysis cannot be interpreted with one plot. Instead, we provide an interactive surface, where the significant changes are available on each of the 64 channels in 10 ms-time bins (Supplementary Data 1).

**Figure 4:**
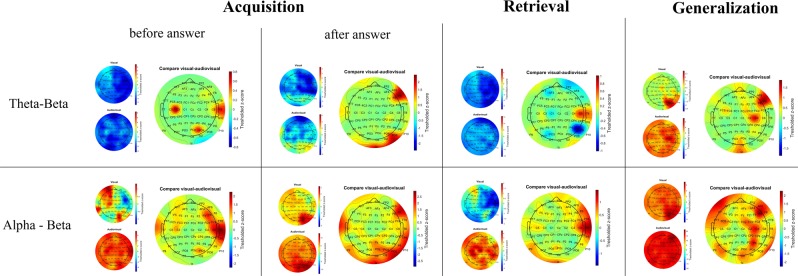
Topographical representation of the cross-frequency theta-beta and alpha-beta coupling results during the visual and audiovisual task. In the smaller topographical plots, the significant difference between SI-values of the given phase and the background activity is shown. The red color indicates where the SI was significantly higher during the given phase compared to baseline-activity, and the blue color indicates where the SI in the given phase was significantly lower compared to baseline activity. On the larger topographical plots, we present the significant difference between the visual and audiovisual paradigm. Here, the red color indicates that the power of that specific frequency band in the given phase of the paradigm was significantly higher during the audiovisual task compared to the visual task, where the blue color indicates the opposite.

In the cross-frequency coupling results, the group-level statistical differences between the mean synchronization indices of the visual and Audio-visual paradigm are presented in each frequency band, and in each phase of the paradigm. Also, detailed descriptions of the SI-value changes of each phase of the visual and Audio-visual paradigm compared to the SI-value of the baseline activity are presented. Significant changes of the cross-frequency coupling in each channel in different phases of the paradigm will be presented using the *topoplot* function of EEGLab. For plotting purposes, only significant changes (i.e. where the Z-scores were >1.69) are presented on the plots (Fig. 4). In the smaller topographical plots, the significant difference between SI-values of the given phase and the background activity is shown. The red color indicates where the SI was significantly higher during the given phase compared to baseline-activity, and the blue color indicates where the SI in the given phase was significantly lower compared to baseline activity. On the larger topographical plots, we present the significant difference between the visual and audiovisual paradigm. Here, the red color indicates that the power of that specific frequency band in the given phase of the paradigm was significantly higher during the audiovisual task compared to the visual task, where the blue color indicates the opposite. As we found that the highest changes in the individual comodulogram occurred at the modulating frequency band 8–15 Hz and the modulated frequency band at 31–45 Hz, our results will indicate the group level results found in that frequency range.

**Figure 5:**
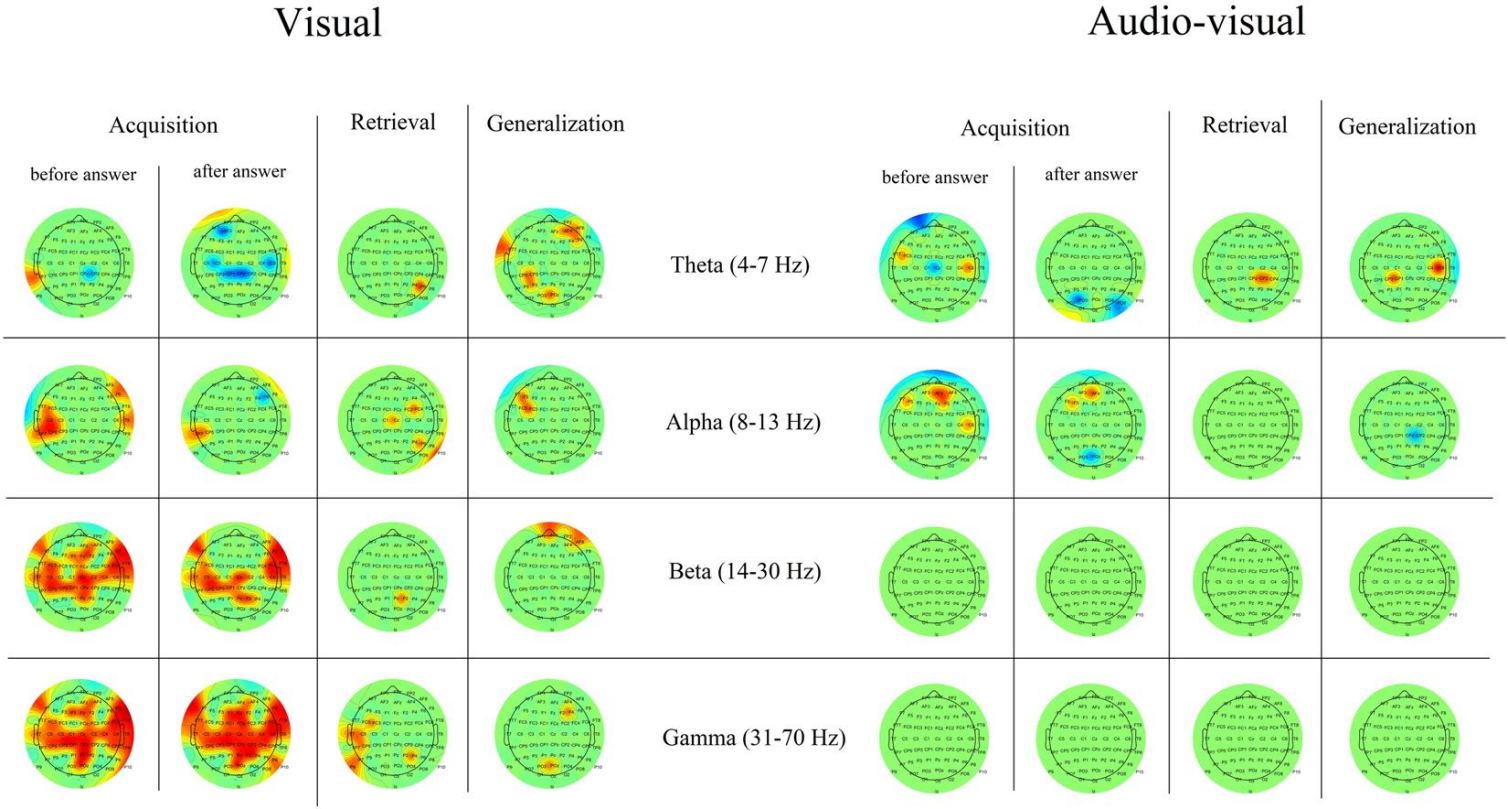
Topographical representation of the power-performance correlation in each phase of the paradigm, during the visual and audiovisual task. Significant correlations (i.e. where the t-values were >2.583) are presented in each phase of the visual and audiovisual paradigm. For plotting purposes, only significant correlations between the performance in the psychophysical test and the cortical power changes are plotted on the topographical figures.

In the power-performance-correlation results, significant correlations (i.e. where the t-values were >2.583) are presented in each phase of the visual and audiovisual paradigm. For plotting purposes, only significant correlations (i.e. Z-score > 1.69) between the performance in the psychophysical test and the cortical power changes (Fig. 5) are plotted on the topographical figures.

### Performance in the visual and the Audio-visual psychophysical tests

**Figure 6:**
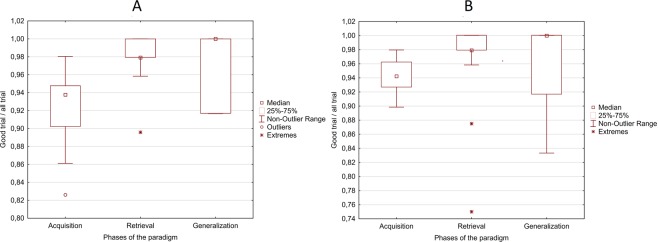
Box plots of the correct trial ratios in each phase of the paradigm during visual (**A**) and Audio-visual (**B**) acquired equivalence learning test.

The mean correct trial ratios (correct trials/all trials) during different phases of the visual acquired equivalence test were as follows: 0.92 in the acquisition phase (range = 0.83–0.98, SD ± 0.04), 0.98 in the retrieval phase (range = 0.9–1, SD ± 0.02), and 0.98 in the generalization phase (range = 0.92–1, SD ± 0.04), (Fig. 6A). Repeated measure analysis of variance (ANOVA) revealed significant difference in the correct trial ratios (F = 20.87, p < 0.001), and Tukey post-hoc analysis revealed that the correct trial ratio in the acquisition phase was significantly lower compared to the retrieval and generalization phases (p < 0.001). The correct trial ratios did not differ significantly between the generalization and retrieval phase (p = 0.992).

The mean correct trial ratios during different phases of the Audio-visual acquired equivalence test were as follows: 0.94 in the acquisition phase (range = 0.90–0.98, SD ± 0.02), 0.98 in the retrieval phase (range = 0.88–1, SD ± 0.03), and 0.97 in the generalization phase (range = 0.83–1, SD ± 0.05), (Fig. 6B). Repeated measure ANOVA revealed significant difference in the correct trial ratios (F = 7.49, p = 0.002), and Tukey post-hoc analysis revealed that the correct trial ratio in the acquisition phase was significantly lower compared to the retrieval (p = 0.002) and generalization phases (p = 0.019). The correct trial ratios did not differ significantly between the generalization and retrieval phase (p = 0.709).

### Group-level analysis of the time-frequency power spectra in the visual and Audio-visual associative learning paradigms

We interpret our EEG results in each frequency band by showing the cortical areas and the corresponding channels where we found significant differences between the power spectra of the two paradigms. Because of the huge amount of data, our detailed results cannot be interpreted with one plot. Instead, we provide an interactive surface where each significant difference is presented in all 64 channels and in each 10 ms-time section (Supplementary Data 1). Additionally, we provide summarizing pictures and a table of our results (see in Figs 1–3 for Acquisition, Retrieval, Generalization, respectively and Table 1).

**Table 1:** Table of the statistics of the most important differences between the visual and audiovisual time-frequency results.

Condition	Frequency band	Time bounds	Cortical region	Power in visual paradigm				p-value	Power in audiovisual paradigm			
				mean	std	min	max		mean	std	min	max
Acquisition	Theta	−400ms −170ms	Frontal	−0,042	0,459	−3,698	2,012	<0.001	0,118	0,500	0,000	2,814
Acquisition	Theta	0 ms 400 ms	Frontal Parietooccipital	−0,023	0,655	−6,954	5,212	<0.001	0,078	0,494	−1,943	6,794
Acquisition	Alpha	−350ms −170ms	Occipital	−0,278	1,159	−6,644	0,000	<0.001	0,012	0,140	−1,386	1,041
Acquisition	Gamma	0 ms 500 ms	Parietal	0,080	0,360	−0,842	3,491	0.005	0,093	0,417	−0,496	4,472
Retrieval	Theta	−500 ms 0 ms	Temporal Frontal	−0,020	0,276	−3,549	2,006	<0.001	0,066	0,383	−1,871	3,718
Retrieval	Alpha	−500 ms 0 ms	Parietooccipital	−0,253	1,058	−8,308	0,000	<0.001	−0,036	0,222	−2,324	0,971
Retrieval	Beta	−500 ms 0 ms	Occipital Parietooccipital	−0,093	0,470	−5,524	1,110	<0.001	0,027	0,276	−3,610	2,994
Retrieval	Gamma	−500 ms 0 ms	Frontal + Parietooccipital	−0,041	0,302	−4,662	2,088	<0.001	0,026	0,270	−3,205	4,133
Generalization	Theta	−500 ms 0 ms	Frontal + Parietooccipital	−0,107	0,677	−8,810	2,529	<0.001	0,009	0,356	−3,022	3,157
Generalization	Alpha	−500 ms 0 ms	Occipital + Parietooccipital	−0,245	1,065	−8,110	0,381	<0.001	−0,033	0,409	−4,785	3,102
Generalization	Beta	−500 ms 0 ms	Parietooccipital	−0,066	0,438	−6,089	3,068	<0.001	0,017	0,347	−5,557	3,933
Generalization	Gamma	−500 ms 0 ms	Frontal + Parietooccipital	−0,038	0,376	−5,521	3,490	<0.001	0,026	0,346	−4,803	4,537

We provide a summarizing table of the descriptive statistic of the normalized powers in different frequency band and time-window identified in the interactive surface provided in the Supplementary Data 1. The p-value between the visual and audiovisual task was calculated with Mann-Whitney test.

### Acquisition phase

For the graphical interpretation of our time-frequency results in the acquisition phase see Fig. 1.

The Mann-Whitney test revealed that the power of the theta band was significantly higher during the audiovisual paradigm (mean = 0.118 dB, STD = 0.5 dB, Range = 0 dB 2.814 dB) compared to the visual paradigm (mean = −0.042 dB, STD = 0.459 dB, Range = −3.698 dB 2.012 dB) over the frontal channels, 400 ms to 170 ms before the answer. After the answer (from 0 ms to 400 ms after the answer), the power of the theta band was significantly higher (p < 0.001) in the audiovisual paradigm (mean = 0.078 dB, STD = 0.494 dB, Range = −1.943 dB 6.794 dB) compared to the visual paradigm (mean = −0.023 dB, STD = 0.655 dB, Range = −6.954 dB 5.212 dB) not only over the frontal but over the parietooccpital channels, too.

In case of the alpha frequency band we found that the power was significantly lower (p < 0.001) during the visual paradigm (mean = −0.278 dB, STD = 1.159 dB, Range = −6.664 dB 0 dB), than in the audiovisual one (mean = 0.012 dB, STD = 0.140 dB, Range = −1.386 dB 1.041 dB) over the occipital channels, 350 ms to 170 ms before the answer.

We observed no significant difference in the beta power between the visual and the audiovisual paradigm.

The power of the gamma band was significantly higher (p = 0.005) during the audiovisual paradigm (mean = 0.093 dB, STD = 0.417 dB, Range = −0.496 dB 4.472 dB) than in the visual one (mean = 0.08 dB, STD = 0.36 dB, Range = −0.842 dB 3.491 dB), over the parietal channels, starting from 0 ms until 500 ms after the given answer.

### Retrieval phase

For the graphical interpretation of our time-frequency results in the retrieval phase see Fig. 2.

The Mann-Whitney test revealed that the power of the theta band was significantly higher (p < 0.001) in the audiovisual paradigm (mean = 0.066 dB, STD = 0.383 dB, Range = −1.871 dB 3.718 dB) than in the visual paradigm (mean = −0.02 dB, STD = 0.276 dB, Range = −3.549 dB 2.006 dB) over the temporal and frontal channels, 500 ms to 0 ms before the answer.

In case of the power of the alpha frequency band we found that it was significantly lower (p < 0.001) during the visual paradigm (mean = −0.253 dB, STD = 1.058 dB, Range = −8.308 dB 0 dB) than in the audiovisual paradigm (mean = −0.036 dB, STD = 0.222 dB, Range = −2.324 dB 0.971 dB) over the parietooccipital channels, from 500 ms before the answer.

The power of the beta frequency band was significantly higher (p < 0.001) during the audiovisual paradigm (mean = 0.027 dB, STD = 0.276 dB, Range = −3.61 dB 2.994 dB) than in the visual paradigm (mean = −0.093 dB, STD = 0.47 dB, Range = −5.524 dB 1.11 dB), over the occipital and parietooccipital channels, from 500 ms before the answer.

The power of the gamma band was significantly higher (p < 0.001) during the audiovisual paradigm (mean = 0.026 dB, STD = 0.27 dB, Range = −3.205 dB 4.133 dB) compared to the visual paradigm (mean = −0.041 dB, STD = 0.302 dB, Range = −4.662 dB 2.088 dB), over the frontal and parietooccipital channels, starting from 500 ms before the answer.

### Generalization phase

For the graphical interpretation of our time-frequency results in the generalization phase see Fig. 3.

The Mann-Whitney test revealed that the power of the theta band was significantly higher (p < 0.001) during the audiovisual paradigm (mean = 0.009 dB, STD = 0.356 dB, Range = −3.022 dB 3.157 dB) than in the visual one (mean = −0.127 dB, STD = 0.677 dB, Range = −8.810 dB 2.529 dB) over the frontal and parietooccipital channels, from 500 ms before the answer.

The power of the alpha frequency band was significantly lower (p < 0.001) during the visual paradigm (mean = −0.245 dB, STD = 1.065 dB, Range = −8.11 dB 0.381 dB), compared to the audiovisual paradigm (mean = −0.033 dB, STD = 0.409 dB, Range = −4.785 dB 3.102 dB) over the occipital and parietooccipital channels, from 500 ms before the answer.

The power of the beta frequency band was significantly higher (p < 0.001) during the audiovisual paradigm (mean = 0.017 dB, STD = 0.347 dB, Range = −5.557 dB 3.102 dB) compared to the visual paradigm (mean = −0.066 dB, STD = 0.438 dB, Range = −6.089 dB 3.068 dB), over the parietooccipital channels, starting from 500 ms before the answer.

The power of the gamma band was significantly higher in the audiovisual paradigm (mean = 0.026 dB, STD = 0.346 dB, Range = −4.803 dB 4.537 dB) than in the visual paradigm (mean = −0.038 dB, STD = 0.376 dB, Range = −5.521 dB 3.49 dB), over the frontal and parietooccipital channels, starting from 500 ms before the answer.

#### Correlation between TF-power and psychophysical performance

To reveal the significant TF-power that correlated with the psychophysical performance, we calculated the correlation between the mean power of different frequency bands and performance in the different phases of the paradigm (Acquisition – before and after the given answer – Retrieval, Generalization). See detailed description of the calculations in the Materials and Methods section. In this section we summarize the significant differences in the regions in different frequency bands during the different phases of the paradigm. In the topographical figures we present the significant correlation coefficients found in different phases of the paradigm (Fig. 5).

#### Visual associative learning paradigm

We found a positive correlation between the power of the higher frequency oscillations (beta, gamma) during the acquisition phase over the parietal and temporal channels before and after the answer.

In the alpha band, we found positive correlations between the power over the temporal channels before and after the answer and performance.

In the theta band power, we found a positive correlation between the performance and power before the answer over the temporal channels. However, we found that the power of theta band over the parietal channels after the answer was negatively correlated with performance.

During the retrieval phase of the visual-associative learning paradigm, we found that power of the gamma band over the temporal channels positively correlated with performance. Concerning the lower frequency bands, their power over the parietooccipital channels positively correlated with performance.

In case of the generalization phase, we observed positive correlation between the psychophysical performance and the power of beta band over the frontal channels.

In case of the alpha band, we observed positive correlation over the frontal channels, and in case of the theta band we found positive correlation over the frontotemporal and parietal channels.

#### Audiovisual associative learning paradigm

In the acquisition phase of the audiovisual paradigm we found no significant correlation between the power of the higher frequency band (beta, gamma) and the psychophysical performance.

We observed positive correlation between the power of the alpha band before the answer over the frontal and frontotemporal channels and performance. After the answer we found, that the power of the alpha band positively correlated over the frontal channels, and negatively correlated over the parietooccpital channels.

We observed negative correlation over the frontal channels and positive correlation over the temporoparietal channels between performance and the power of the theta band before the answer. After the answer we found, that the power of the theta band negatively correlated over the parietooccipital channels with performance.

In the retrieval phase of the audiovisual paradigm we found, that the power of the theta band over the parietal channels positively correlated with the psychophysical performance. We did not find any significant correlations between the power of other frequency bands and the psychophysical performance.

In the generalization phase we found, that the power of the alpha band over the parietooccipital negatively correlated with performance. We also found that the power of the theta band over the parietotemporal channels positively correlated with performance.

### Cross-frequency coupling in the visual and Audio-visual associative learning paradigms

During the acquisition phase of the paradigm, we found a significant decrease in cross-frequency theta-beta coupling compared to baseline-activity both in the case of the visual and the audiovisual paradigm before the given answer. After the given answer, we found significant SI-elevation compared to baseline activity over the occipital-parietooccipital channels (Iz, PO8), which was higher during the audiovisual paradigm.

Regarding the alpha-beta coupling, we found a significant increase of the SI-values before the answer over the whole scalp in the case of the audiovisual paradigm, and over the left frontal-frontotemporal (FP1, AF3, AF7, F1, F3, F5, F7, FC1, FC3, FC5) and parietooccipital-occipital (PO4, O2) channels. Comparing the visual and the audiovisual paradigm, before the given answer in the acquisition phase, we found that the SI-values were significantly higher over the temporal (T7, T8, TP8, C6) and frontotemporal (FC6, F6) channels during the audiovisual paradigm.

After the answer, we found that alpha-beta coupling was significantly higher compared to the baseline activity both in the visual and the audiovisual paradigm, predominantly over the parietooccipital-occipital (PO4, PO8, O2, Oz) channels, which was higher during the audiovisual paradigm.

During the retrieval phase of the paradigm, we observed significant theta-beta coupling decrease over the whole scalp during both the visual and audiovisual paradigm. Comparing the visual and the audiovisual paradigm, we found that the SI-decrease was higher over the right parietooccipital (P8, P6) channels during the visual paradigm.

For the alpha-beta coupling, we observed a significant decrease of the SI-values compared to baseline activity over the right temporal, frontotemporal and parietoocciptal channels during the visual paradigm. We observed significant alpha-beta cross-frequency coupling increase over the whole scalp during the audiovisual paradigm. Comparing the visual and the audiovisual paradigm, we found that the SI-values were higher over the right temporal-frontotemporal (T8, C6, FT8, F8) and parietooccipital-occipital (Iz, P9) channels during the audiovisual paradigm.

During the generalization phase, we found significant theta-beta coupling over the parietooccipital-occipital channels (PO8, PO4, P2, P4, P6, P8) during the visual paradigm. We found that the theta-beta coupling was significantly higher compared to baseline activity over the whole scalp during the audiovisual paradigm. Comparing the visual and audiovisual theta-beta coupling strength, we found that the SI-values were significantly higher over the frontotemporal (C4, F8, F6) and occipital (Iz, Oz, POz) channels during the audiovisual paradigm.

In the case of the alpha-beta coupling, we found the same pattern of SI-value increase that was observed in the theta-beta coupling during the generalization phase.

## Discussion

The present study analyzed the EEG correlates in a visually-guided and an Audio-visually (bimodal or multisensory) guided acquired -equivalence learning tasks. To our knowledge, this is the first study to address the comparison of the cortical power spectra and their changes in a unimodal visual and a multisensory associative learning task. The major finding of the study is that the cortical activity depends critically on the phase of the paradigm, and some changes in cortical powers are characteristic to unimodal visual and multisensory Audio-visual tasks. In general, during the audiovisual paradigm, the power changes of the event-related low and high-oscillations were higher compared to the visual paradigm, but the psychophysical performance of the acquisition phase only correlated with the power of different frequency bands during the visual paradigm. On the other hand, while the power changes of the event-related oscillations were higher during the audiovisual paradigm, the performance did not depend on the power of different oscillations, and the strength of the cross-frequency coupling was higher. Furthermore, the performance of the acquisition phase seems to be more connected to the strength of the alpha-beta coupling during the audiovisual paradigm. We are convinced that the cortical power differences in the two paradigms cannot be the result of having previously completed the first task (precondition), hence the order of the two paradigms (visual and Audio-visual) varied randomly across subjects.

The role of multimodal cues in associative learning has been widely investigated (for review see^40^). The psychological studies provided evidence, that multisensory working memory improves recall for cross-modal objects compared to modality-specific objects^41,42^, working memory capacity is higher for cross-modal objects under certain circumstances^43^ and visual and auditory information can interfere with each other^44^. While former studies revealed mainly cortical areas are involved in associative learning^45,46^, only few electrophysiological studies showed the functional basis of the multisensory integration^47^, and to our knowledge, our study is the first that describes the role of different oscillations in multisensory integration during learning.

The performance of the investigated population in the psychophysical test (acquisition error ratio, retrieval error ratio, generalization error ratio) was in the same range as that of the earlier investigated healthy controls of neurological and psychiatric patients^2,4,6,48^. Based on this we are strongly positive that the electrophysiological results showed here are representative.

One of the common findings both in the visual and the Audio-visual paradigm is the increased theta band activity in the parietooccipital, frontal midline, and prefrontal areas during the acquisition and retrieval phases. Frontal midline theta activity has been widely investigated (for review, see^49^), and its contribution seems to be obvious to internally-guided cognitive tasks that require no external responses^50–52^. A more general interpretation of the increased theta power in the frontal cortex could be the coordinated reactivation of information represented in visual areas. This was also found in single-unit recordings in primate V4^53^ as well as LFP-synchronisation between the prefrontal cortex and V4^54^. Regarding our findings, we hypothesize that the initial acquisition phase of the task requires more repeated reactivation of the cortical areas where the stimulus is processed. We also assume (based on the power-performance correlation we observed) that the better the associations are encoded, the more enhanced theta activity can be observed in the frontal midline areas.

The earlier findings of human electrophysiological studies indicated the role of the alpha band in visual^55,56^ as well as Audio-visual processing^57^. Moreover, Hanslmayr and his colleagues^58^ found that the performance in processing stimuli is more likely to be linked to decreased power in the alpha band. Another function that has been attributed to alpha activity is a mechanism of sensory suppression, thus functional gating of information in the task-irrelevant brain areas^59,60^. Indeed, our findings suggest that the initial parts of the trials (0–50 ms) are coupled to decreased alpha power, which were then followed by an increase of it in the visual (occipital) and Audio-visual (parietooccipital) cortical areas. These together suggest that after a rapid processing of the cue image and sound, the threshold of the visual/Audio-visual cortical areas for external stimuli becomes higher, allowing the information to be encoded (or retrieved) internally.

In the case of the beta frequency range, there is a growing evidence that enhanced beta oscillations appear in patients with Parkinson disease (PD)^61^. A number of investigations found that a power increase of the beta frequency band correlates with the severity of parkinsonian motor symptoms such as akinesia and rigidity^62,63^. As a result, beta oscillations are currently investigated as a potential biomarker tracking the effectiveness of deep brain stimulation treatment of PD patients^64^. Furthermore, deep brain stimulation of the subthalamic nucleus at beta frequencies worsens motor symptoms in PD patients^65^. One of the main functions of the basal ganglia is to contribute to associative learning by the trial-and-error method^66,67^. It is well-known that in PD (along with other deficits in which basal ganglia are affected) this learning mechanism is reduced^33,34^. Having seen in our results that a robust decrease of beta-power occurred in all phases (acquisition, retrieval and generalization phases) of both the visual and Audio-visual learning tasks, one may consider that this cortical power density decrease in the beta band is a necessary cortical outcome of the normal action of the basal ganglia in visual and Audio-visual associative learning

The gamma frequency band plays an important role in memory processes^68^ as well as other cognitive processes, such as word learning, reading, and expectancy^36,37^. We observed a power increase during the acquisition phase of the task in the frontal cortex and in associative cortical areas connected to the modality of the presented stimuli (i.e. the occipital cortex in the visual task, the parietotemporal areas in the Audio-visual task). Thus, we hypothesize that the acquisition phase of the learning paradigm needs strong cortical contribution. The power differences between the acquisition phase of the visual and Audio-visual learning paradigm suggest stronger cortical contribution to the multisensory learning task. On the other hand, this increase in the gamma power was not detectable in the retrieval and the generalization phases of the paradigm. The explanation for this could be that the already-learned acquisitions were already transmitted to the hippocampus and the application of the earlier acquisitions does not need strong cortical activation in the gamma band. There is also evidence that a decrease in the gamma power of the local field potential correlates with performance and attention by selectively gating sensory inputs^69,70^ We assume that the decrease of the gamma power we found during the task over the cortical areas where the stimulus was processed (i.e. occipital areas in the visual and parietotemporal in the Audio-visual task) could be beneficial in memory encoding and retrieval by filtering irrelevant external stimuli.

Calculating the synchronization indices, we found increased coupling between theta and alpha/beta in each phase of both the visual and the Audio-visual task, which are in accordance with earlier studies that emphasize the role of theta-gamma/beta coupling in memory processes^40,41^ as well as the alpha gamma/beta coupling in visual perception^42,43^. We also found that this synchronization was significantly stronger during each phase of the Audio-visual task than in the visual paradigm. Furthermore, during the retrieval and generalization phases, we found that the synchronization between theta-beta and alpha-beta was significantly stronger in the audiovisual task. We argue that the audiovisual paradigm required stronger synchronized cortical contribution in all phases of the task because of the cross-modal integration of the visual and auditory stimuli. Furthermore, as the power of the cortical oscillations increased more during the visual task compared to the Audio-visual task, this indicates that the multimodal (audiovisual) associations require less, but more synchronized activation of the cortex.

Optimal performance in the acquisition phase of the learning paradigms appears to depend mainly on the integrity of the basal ganglia, whereas performance in the test phase (both retrieval and generalization) has been linked to the integrity of the hippocampal region^2,71,72^. It is also known that both fundamentally-involved structures, the basal ganglia and the hippocampi, are involved in attention processes (for a reviews, see^73^). At the behavioral level, multisensory integration could be dependent on the level of attention and is not an automatic, unconscious process^74^. A multisensory task seems to be more complex and probably needs more attention from the participants. However, our psychophysical results show no significant differences between the performances in the visual and the multisensory tasks. In summary, although the role of attention cannot be excluded in the psychophysical learning test, the same level of performance in the unimodal visual and multimodal (Audio-visual) learning paradigms contradicts the assumption that attention contributes significantly to the differences in cortical activation patterns.

We can conclude that the changes in the power of the different frequency-band oscillations were more critical during the visual paradigm. On the other hand, the encoding and the retrieval part of the bimodal, Audio-visual task required more strongly synchronized cortical activity than those of the visual one. The two former statements are probably due to the fact that in the case of the multisensory associations the investigated memory processes (encoding, retrieval, and generalization) require less prominent, but more synchronized cortical activation, while the unimodal associations require more prominent and less synchronized cortical activity during the same memory processes. These findings further emphasize the effect of multimodal integration during associative learning and memory processes.

## Materials and Methods

The EEG data of 23 adult healthy young adults were recorded (12 females, 11 males, mean age: 26 years, range = 18–32). The participants were free of any ophthalmological or neurological conditions, and they were tested for parachromatism with the PseudoIsochromatic Plate Color Vision Test. The participants were recruited on a voluntary basis. The potential subjects were informed about the background and goals of the study, as well as about the procedures involved. It was also emphasized that, given the lack of compensation or any direct benefit, the participants were free to quit at any time without any consequence (no one did so). Those who decided to volunteer signed an informed consent form. The study protocol conformed to the tenets of the Declaration of Helsinki in all respects, and was approved by the Medical Ethics Committee of the University of Szeged, Hungary (Number: 50/2015-SZTE). The datasets generated and analyzed during the present study and the Matlab codes, that were used in the analytical process connected to this study are available in the Supplementary Data 2.

### Visual associative learning test

The testing software (described in earlier studies and originally written for iOS^2^) was adapted to Windows. It was coded in Assembly for Windows and translated into Hungarian, with the written permission of the copyright holder. The paradigm was also slightly modified to reduce the probability of completing its acquisition phase by mere guessing (see below). The tests were run on a PC. The stimuli were displayed on a standard 17- inch CRT monitor (refresh rate 100 Hz) in a quiet room separated by a one-way mirror from the recording room. Participants sat at a 114 cm distance from the monitor. One participant was tested at a time and no time limitation was set. The test was structured as follows: in each trial of the task, the participants saw a face and a pair of fish of different color, and had to learn through trial and error which fish was connected with which face (Fig. 1). There were four faces (A1, A2, B1, B2) and four possible fish (X1, X2, Y1, Y2), referred to as antecedents and consequents, respectively. In the initial, acquisition stages, the participants were expected to learn that when A1 or A2 appears, the correct answer was to choose fish X1 over fish Y1; given face B1 or B2, the correct answer was to choose fish Y1 over fish X1. If the associations were successfully learned, participants also learned that face A1 and A2 were equivalent with respect to the associated fish (faces B1 and B2 likewise). Next, participants learned a new set of pairs: given face A1, they had to choose fish X2 over Y2, and given face B1, fish Y2 over X2. This was the end of the acquisition phase. To this point, the computer provided feedback about the correctness of the choices, and six of the possible eight fish-face combinations were taught to the participants. In the following phases (retrieval and generalization), no feedback was provided. Beside the already-acquired six pairs (tested in the retrieval phase) the hitherto not shown two pairs were also presented, which were predictable based on the learned rules (tested in the generalization phase). Having learned, that faces A1 and A2 are equivalent, participants were expected to generalize from learning that if A1 goes with X2, A2 also goes with X2; the same holds for B2 (equivalent to B1) and Y2 (equivalent to B1). During the acquisition stages, new associations were introduced one by one, mixed with trials of previously-learned associations. The subjects had to achieve a certain number of consecutive correct answers after the presentation of each new association (4 after the presentation of the first association, and 4, 6, 8, 10, 12 with the introduction of each new association, respectively) to be allowed to proceed. In order to minimize the repetition effect in the acquisition phase, the last, 12-answer trial of the acquisition phase were set to be the part of the retrieval phase. This resulted in an elevated number of the required consecutive correct trials compared to the original paradigm, which made getting through the acquisition phase by mere guessing less probable. Similarly, in the test phase there were 48 trials (12 trials of new and 36 trials of previously-learned associations), as opposed to the 16 trials of the original paradigm.

### Audio-visual associative learning test

**Figure 7:**
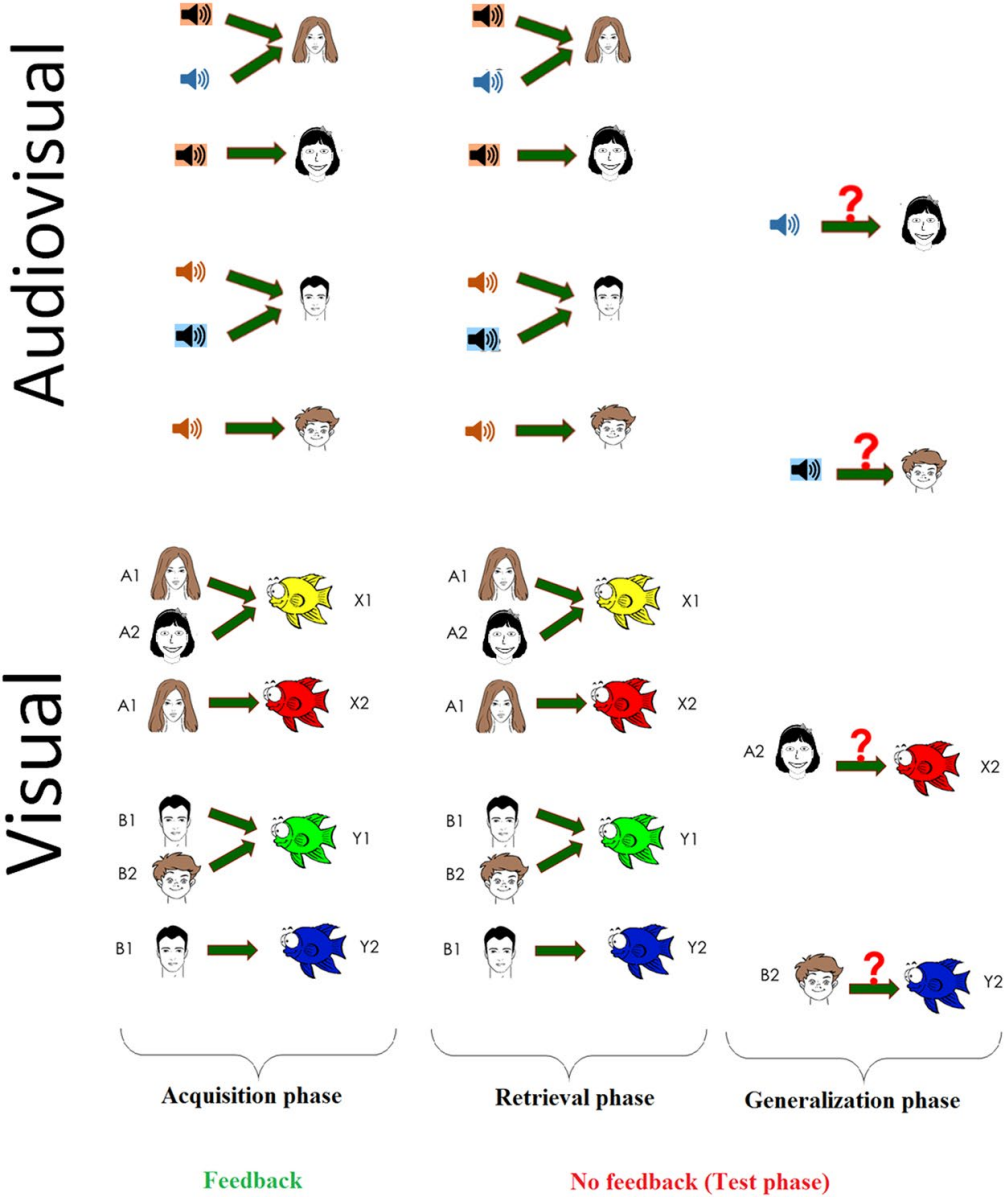
Graphic overview of the unimodal visual (lower panel) and the bimodal Audio-visual (upper panel) acquired equivalence paradigms.

We developed the Audio-visual (multisensory or bimodal) guided acquired equivalence learning test. The structure of the paradigm was the same as of the visual associative learning test, with the difference that the four antecedents were four sounds (A1, A2, B1, B2) and the consequents were the same four faces as in the visual associative learning paradigm. The main task of the participants was to determine from trial to trial which of the two given faces corresponds to the sound heard at the beginning of the trial. The sounds of the paradigm were a female voice saying “Hello”, the sound of a guitar, the sound of a motorcycle, and the sound of a cat. Each sound lasted less than 1 sec. The category rule implemented in the four faces (i.e. sex, age, hair color) was the same as in the visual paradigm, so similarity across the sounds was not important, but the similarity across the four faces was the same as in case of the visual paradigm. During the acquisition phase, six of the possible eight sound-face combinations were learned. During the test phase, no feedback was provided anymore, but beside the already-acquired six pairs (learned in the retrieval phase), the hitherto not shown last two pairs were also presented (generalization phase). For visual representation of the two task, see Fig. 7.

### Data acquisition

Sixty-four channel EEG recordings were performed using a Biosemi ActiveTwo AD-box with 64 active electrodes. Actiview software was used to set up the parameters and record the EEG-data (Biosemi B.V., The Netherlands). The sampling rate was 2048 Hz. Electrode offsets were all kept within normal and acceptable ranges. Raw signals were recorded on the computer that controlled the psychophysical learning task. The stimulating software generated trigger signals to indicate the beginning of each trial. These trigger signals were recorded on an additional (sixty-fifth) channel. In order to obtain the baseline activity, one-minute-long resting state activities were recorded before and after testing the visual and Audio-visual associative learning test. The order of the two tests (visual and Audio-visual associative learning test) varied randomly across volunteers.

### Preprocessing

The raw EEG data was first high-pass filtered (the filtering method used a 2 Hz highpass, two-way, least-squares FIR procedure, as implemented in the eegfilt.m script included in the eeglab package)), and then re-referenced to the average of the channels. All trials were visually inspected, and those containing EMG or other artefacts not related to blinks were manually removed. Independent components analysis was computed using the EEGLab toolbox for Matlab^75^, and components containing blink/oculomotor artefacts or other artefacts that could be clearly distinguished from brain-driven EEG signals were subtracted from the data. The trials were defined as 500 ms before and after the given answer. In order to minimize the repetition effect in the acquisition phase, the last 12-answer trial of the acquisition phase was set to be the part of the retrieval phase. The subtracted trials did not exceed 1% of all trials. The mean trials of the phases of the paradigm was 54, 50 and 12, in the acquisition, retrieval and generalization phase, respectively. Additionally, noisy channels were interpolated using EEGLab toolbox. Then we used Laplacian fitting to improve the spatial resolution of the recording^76^. After the preprocessing steps, the data was resampled to 256 Hz, to minimize the computational time. This is the reason that in the present study the results of the time-frequency analysis are given in 10 ms-bins.

### Data analysis

All data analysis was performed using Matlab (MATLAB and Statistics Toolbox Release 2018a, The MathWorks, Inc., Natick, Massachusetts, United States.) and Statistica software (Dell Statistica for Windows v13).

### Analysis of the performances in the psychophysical learning tasks

The psychophysical data were analysed in three groups: data from the acquisition phase, data from the retrieval parts of the test phase (i.e. when the participant was presented an already-learned association), and data from the generalization part of the test phase (i.e. previously-not-learned associations). The number of correct and wrong responses were calculated in all phases, as well as the ratio of these to the total number of trials during the respective phase.

### Time-frequency analysis

Time-frequency analysis was performed using a Continous Morlet wavelet convolution (CMW) via FFT algorithm^77^. Firstly, FFT was first performed on one selected channel of the raw data. Then complex Morlet wavelets were created for each frequency (1–70 Hz) on which FFT was also executed. The cycles of the wavelets increased logarithmically as the frequency varied in a linear manner. After that, we calculated the dot product of the given channel’s FFTs and the FFTs of the complex Morlet wavelets at each individual frequency, which yielded 70 complex numbers. Thereafter, the inverse Fast Fourier transform of the results of the dot product showed the alterations of the power in the time domain as follows:

$${K}_{x}=IFFT(fft(C)\cdot fft({W}_{x}))$$

where the *K* is the time-series of the given channel, wavelet-filtered to frequency x, *C* is the time series of all trials of different phases, and *W* is the complex Morlet wavelet in a given frequency x. In order to avoid the edge-artifacts of the Morlet wavelet convolution, the raw data was multiplied five times before the convolution, yielding a two-series-long buffer zone at the beginning and the end of the time-series, which was cut out after the time-frequency analysis. After that, the channel’s data was cut into different phases of the paradigm (baseline, acquisition, retrieval, generalization). The trials were defined as the 500 ms before and after the given answer. As the baseline activity was longer than the compared periods (i.e. the signal belonging to a given condition), the baseline activity was bootstrap-resampled to match the given condition by cutting one-second-long periods randomly from the baseline activity (1–1 minute before and after the first and the last trigger-signal, respectively). The bootstrapping method was the same as described in^78^.The data set for the purposed null-hypothesis (global band) was generated by iteratively calculating the mean difference of the randomized permutation of the power values of a particular channel in a given frequency band in two different phases of the paradigm (Baseline-Acquisition, Baseline-Retrieval, Baseline-Generalization). The Z-scores for each channel were then calculated between the distributions derived from the global band and the mean difference of the power values in a given frequency band between the analysed phase and the baseline activity. Z-scores were corrected by the minimum and maximum point of the null hypothesis distribution (also known as cluster-mass correction^77,79^).

Group-level analysis of the CMW was carried out in the same way as in the individual analysis described above, with the difference that the random permutation was performed across the mean power values of the subjects and not across the power value of each individual trial. The visualized methodological procedures of the above-described permutation-based test can be seen in one of our earlier publications through an individual example^80^. In the interactive surface provided in the Supplementary Material (Supplementary Data 1) only significant Z-scores calculated the above mentioned way are provided. As the data was resampled to 256 Hz, the time-bins in the Supplementary Data are 10 ms.

We identified the time-windows in which we found significant difference between the visual and the audiovisual paradigm, using the interactive surface provided in the Supplementary Material (Supplementary Data 1). After we identified the significant time-windows and the corresponding channels in each frequency band and condition, we additionally tested if the individual normalized powers of the different frequency bands in the selected channels and time-points are significantly different in the visual and the audiovisual paradigm by using Mann-Whitney test. The individual normalized powers were obtained by normalizing each individual time-frequency power in each condition and on each channel to the mean power of the baseline activity in the same channel and same frequency using decibel-normalization. Furthermore, to see the significant differences in the time-domain in the selected channels, we performed permutation-test between the normalized power time series of the visual and audiovisual test.

### Calculation of event-related cross-frequency coupling

Event-related synchronization index (SI) was calculated in order to examine whether the power of the high-frequency oscillations are coupled to the phase of the low-frequency oscillations on the same channel. The calculation method was almost the same as described by Cohen^81^. We will give a detailed description of the calculation of the SI in one phase of the paradigm in one channel’s data referred as raw analytic signal. In the first step, the higher-frequency power time series were extracted from the concatenated trials. This was done by the combination of band-pass filtering and Hilbert transformation. First, we used a narrow band pass to filter the analytic signal to each frequency of beta and gamma band (15–70 Hz). The filtering method used a 4 Hz-width, two-way, least-squares FIR procedure (as implemented in the eegfilt.m script included in the eeglab package). Then we performed Hilbert transformation on the narrow bandpass-filtered epochs. The power time-series was extracted as the squared magnitude of z(t), the analytic signal obtained from the Hilbert transform (power time series: p(t) = real[z(t)]2 + imag[z(t)]2).

Then we band-pass filtered the raw analytic signal to each frequency of the low-frequency range (2–20 Hz, with 4 Hz-width). The phase of the band-pass filtered low- and high-frequency power time series were obtained from the Hilbert transform of the two time-series, respectively.

The synchronization between the phases of the two power time series can be calculated using the synchronization index (SI) as follows:

$$SI={|\frac{1}{n}\times \sum _{t=1}^{n}{e}^{i({{\rm{\phi }}}_{lt}-{{\rm{\phi }}}_{ut})}|}^{2}$$

where *n* is the number of time points, *φ*_*ut*_ is the phase value of the fluctuations in the higher-frequency power time series at time point *t*, and *φ*_*lt*_ is the phase value of the lower-frequency band time series at the same time point. The SI varied between 0 and 1. At SI 0 the phases are completely desynchronized, and at SI 1 the phases are perfectly synchronized.

Significant changes of the cross-frequency coupling at a population level were calculated by comparing the mean synchronization index in a given modulating - and modulated frequency range of the baseline activity and the given phase of the paradigm. The mean SI-values in each phase of the paradigm were then compared using permutation-based statistics, and the resulting Z-scores were corrected by the minimum and maximum point of the null hypothesis distribution (also known as cluster-mass correction^77,79^). See Supplementary Figs 1 and 2 for the graphical interpretation of the calculation of the SI values and their statistical comparison.

### Correlation between performance in the psychophysical test and the power density changes

Correlation between individual performance and power density changes in a given channel and frequency band was also calculated in each phase of the paradigm. Performance was defined as the ratio of the correct (good) trials to all trials, and the individual power changes were defined as the individual Z-scores between the baseline activity’s power density and the given phase’s power density in a given channel in a given frequency band. The Pearson correlation coefficient was calculated using the *‘corr’* function of Matlab. Statistical analysis was performed by calculating the t-score for each correlation coefficient as follows:

$${t}_{ch,fr}={r}_{ch,fr}\ast \sqrt{\frac{n-2}{1-{r}_{ch,fr}^{2}}}$$

where *r* is the correlation coefficient in channel (*ch*) and frequency (*fr*) band, *n* is the number of samples (in this case it was 18), and *t* is the calculated t value in a given channel and frequency band. T-values whose absolute values were smaller than 2.583 (which is the critical t-value if the degree of freedom is 16 and the significance level is 0.01) were set to 0. The corrected t-values in different frequency bands in each phase of the paradigm were plotted to a topographical map using the ‘*topoplot’* function of EEGLab.

## Supplementary information

Supplementary material
