# Supplementary material for: Power-spectra and cross-frequency coupling changes in visual and Audio-visual acquired equivalence learning
Source: Sci Rep. 2019 Jul 1;9:9444. doi: 10.1038/s41598-019-45978-3 (PMC6603188; doi:10.1038/s41598-019-45978-3)
Supplement: Supplementary file 1 — Supplementary material [file 41598_2019_45978_MOESM1_ESM.docx]

Power-spectra and cross-frequency coupling changes in visual and audio-visual acquired equivalence learning

**András Puszta^1*^, Ákos Pertich^1^, Xénia Katona^1^, Balázs Bodosi^1^, Diána Nyujtó^1^, Zsófia Giricz^1^,** **Gabriella Eördegh^2^, Attila Nagy^1**^**

1: Department of Physiology, Faculty of Medicine, University of Szeged, Dóm tér 10, Szeged H-6720, Hungary

2: University of Szeged, Faculty of Dentistry, Department of Operative and Esthetic Dentistry, Tisza Lajos körút 64, Szeged, Hungary

*: puszta.andras@med.u-szeged.hu

**: nagy.attila.1@med.u-szeged.hu

# Supplementary Data 1

Supplementary Data is provided for the group-level time-frequency results. The standalone Matlab application (Created by Matlab 2018a and compiled with Matlab Compiler) can be found at the Open Science Foundation Data Repository in the following link: <https://osf.io/8jy2s/?view_only=94c27ebd247d45748330733b558eca3b>

The interactive surface consist of two main panel, the upper and the lower panel, in three main columns, that correspond to the different phases of the acquired equivalence paradigm (Acquisition, Retrieval, Generalization). In the upper panel, the topographical representation of the significant power-changes can be seen. At the lower panel, one channel’s significant changes can be seen over the time. The time-frequency plots correspond to the same phase as the topographical plot above them. The reader can change manually the desired channel at the drop box over each time-frequency plot. The specific time-point at which significant changes plotted on the topograhical plots can also be changed with the slider under the lower panel. The frequency band of the topographical representation can be changed in the ‘Menu’ section (upper-left corner). Furthermore, results in the different tasks (visual, audiovisual), as well as the comparison of the two task can be visualized by changing the ‘Analysis mode’ at the Menu.

Colour representation of the figures: Inspecting the visual or the audiovisual task, the red color indicates where the power was significantly higher during the given phase compared to baseline-activity, and the blue color indicates where the power in the given phase was significantly lower compared to baseline activity. On the other hand, comparing the visual and audiovisual time-frequency results the red color indicates that the power of that specific frequency band in the given phase of the paradigm was significantly higher during the audiovisual task compared to the visual task, where the blue color indicates the opposite.

**Supplemetary Data 2: Data, Matlab codes**

In order to increase the transparency, we provide the Matlab codes that were used in the study as well as the pre-processed data. The datasets in each folder corresponds to each participant. Each folder contains a mat-file, and an xls-file (Event file). The datasets in the .mat-files are generated using EEGlab after we removed the artefacts from the raw data, with the methodological steps provided in the main Manuscript.

The Matlab codes are separated in different folders, according to the analytical steps, respectively: TF-analysis, cross-frequency coupling and power-performance correlation. In each folder, the main script is the one, which starts with “_Revision”, and the script that responsible for the group-level analysis starts with “Results_”.

The data can be found in the at the Open Science Foundation Data Repository in the following link:

<https://osf.io/5a9yx/?view_only=0ad72eb2e9f54386a7d1857198a388a3>

And the Matlab codes in the following link:

<https://osf.io/ge45u/?view_only=6ec03b66a09546d6865ecb525b99f998>

# Supplementary methods

## Calculating the synchronisation index


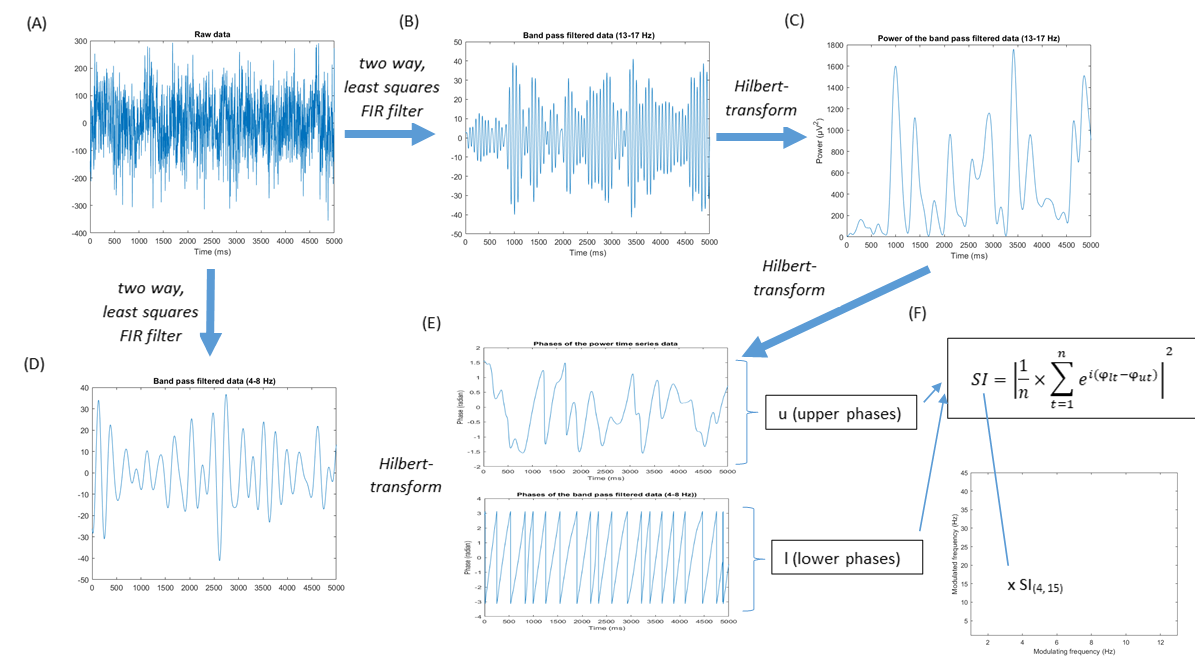


**Supplementary Figure 1:** Graphic overview of the cross-frequency coupling method (based on Cohen MX, 2008). The figure demonstrates how we calculated the synchronisation index between the lower and upper frequency oscillations in a 5-sec long EEG-data of the acquisition phase of the visual acquired equivalence test. The raw analytical signal (A) was first band-pass filtered to a 4 Hz-width band centered at a high frequency (which was 15 Hz in this case). We got the power-alterations (C) of the high-frequency band pass filtered data (B) using Hilbert transformation. The phases of the power alteration of the high-frequency band-pass filtered data (E) was calculated using Hilbert transformation. For the lower frequency band phases, firs we band-pass filtered the raw data to a 4 Hz-width band centered at a lower frequency band (which was 4 Hz in this case, D). The phases of lower frequency band (F) was obtained using Hilbert transformation on the lower frequency band-pass filtered data. Having obtained the phases of the higher frequency band-oscillation (E) and a lower frequency band oscillation (F), we could calculate the SI value between the two oscillations (4 Hz for modulating frequency and 15 Hz for modulated frequency in this case, F).

## Calculating significant changes of the cross-frequency coupling


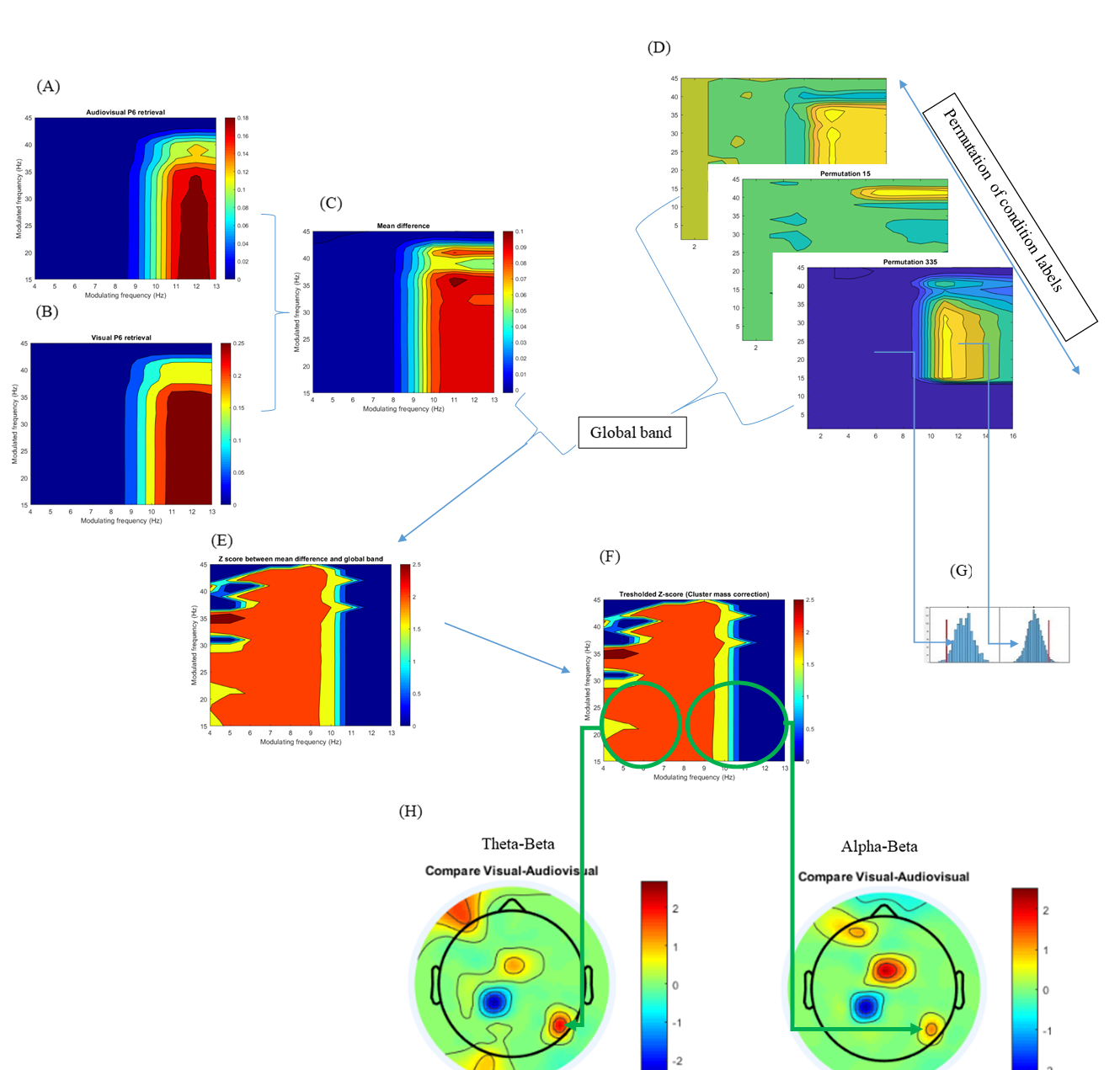


**Supplementary Figure 2:** Graphical overview of calculating the significant changes of the cross-frequency coupling. The figure demonstrates how can be calculated the statistical difference in one channel (in this special case it was P6) between two conditions across subjects. First, we calculated the mean SI values of a given channel in the two condition: in the visual (A) and audio-visual (B) paradigm. Then we calculated the mean difference (C) of them. After that, we iteratively changed the labels of the conditions, and we calculated the mean difference of each permutation (D). After 1000 iterations, we obtained the global band, a comodulogram, whose each datapoint contained 1000 values, thus giving a null-hypothesis distribution. Then we calculated the Z-score between the global band and the mean difference (E). The result was then thresholded to the minimum-maximum distribution of the global band (G). For the clearer interpretation we averaged the Z-scores between alpha-beta and theta-beta, and the mean Z-scores were plotted to a topographical figure (H).

Supplementary Results: Detailed time-frequency results

**Acquisition phase of the associative learning paradigm**

Theta band

During the visual paradigm, we observed increased power over the parietooccipital (PO7, PO8, POz) and the frontal-midline channels (FCz, Fc1, FC2, F2) before the answer. The power elevation was maintained after the answer, and after the answer we also observed a power decrease over occipital (Oz) and parietal (CP1, CP3) channels.

During the audiovisual paradigm, before the answer we observed significant power increase over the frontal-midline (AFz, Fz, FCz, F1, FC1, FC3) channels, and significant power decrease over the occipital (Oz, O2) channels, and from –100 ms over the temporal (T8, TP8) channels. After the answer we found significant power elevation over the occipital-parietooccipital channels (O2, PO1, PO4, PO7, PO8, O2) starting from 50 ms after the answer, and over the frontal channels (AFz, FPz, F2, F4) starting from 150 ms after the answer.

Comparing the power alterations during the visual and audiovisual paradigm, we found that before the answer in the acquisition phase the power was significantly higher in the audiovisual paradigm over the frontal channels (AFz) between -400 ms and -170 ms. However, the power was significantly higher in the visual paradigm 170 ms before the answer over the parietooccipital (PO1, PO4, PO7, PO8) and frontal (FP1, F2, FCz, FC1) channels. After the answer, we found that during the visual paradigm the power was higher over the frontal channels (FC1, FCz).

Alpha band

In the visual paradigm, before the given answer we observed a generalized power decrease compared to baseline activity over the whole scalp, but mainly over the parietal (CPz, CP1, CP2, CP3, CP4) and temporal (CP6, P7, T7, TP8) channels. We also observed a power decrease 30 -300 ms after the answer over the occipital channels (Oz).

During the audiovisual paradigm, we observed significant power decrease over the parietal and temporal areas before and after the answer. 70 ms-170 ms after the answer we also observed significant power elevation over the parietooccipital (PO4, PO8) channels.

Comparing the visual and audiovisual paradigm, we found that power was higher in the audiovisual paradigm over the frontal (FP1, AF3) channels 250 ms-0 ms before the answer. Over the occipital channel (O1), the power was significantly higher from 350 ms to 170 ms before the answer in the audiovisual paradigm. After the answer the power was significantly higher over the parietal (P2, P6), and frontal (F3, AF8) channels in the audiovisual paradigm.

Beta band

In the visual paradigm, we observed power decrease compared to baseline activity over the parietal (CPz, CP1, CP2, CP3) and temporal (CP5, C5, CP6, C6) channels before and after the answer of the acquisition phase. 300 ms after the answer, we found a significant power increase compared to baseline activity over the occipital (O2) channels.

In the audiovisual paradigm, we observed significant power decrease over the parietal (CP2, CPz, C1, Cz, CP3, CP4) and temporal (CP5, CP6) areas before and after the answer (till 300 ms after the answer). After the answer, we also observed significant power elevation compared to baseline activity over the occipital-parietooccipital (POz, PO4, O2, O1, PO3) channels starting from 420 ms after the answer.

Comparing the visual and audiovisual paradigm, we did not observe significant difference between the power changes during the two paradigms before and after the answer.

Gamma band

In the visual paradigm we found significant power decreases over the frontal (AF4, AF3, F4, FC2, FC4) and parietal (CP2 CP4 CP5) channels during the acquisition phase before and after the given answer. After the answer, however, we found a significant power increase over the occipital channels (O2, Oz).

During the audiovisual paradigm, we observed significant power elevation over the occipital-parietooccipital channels (O1, O2, Oz, PO8, O1) and a significant power decrease over the parietal channels compared to baseline activity, before and after the answer.

Comparing the visual and audiovisual paradigm, we found that the power changes of the audiovisual paradigm were significantly higher over the parietal (CP2, CP4, P2) channels after the answer.

**Retrieval phase of the associative learning paradigm**

Theta band

During the visual paradigm, we observed significant power elevations compared to baseline activity over the parietooccipital (PO7, PO8), and frontal-midline (FCz, FC1, Fz, F2, F3, F4) channels.

In the audiovisual paradigm, we observed significant power elevations over the parietooccipital (PO7, PO8), temporal (T8, FT7, T7) and frontal-midline (FCz, FC1, Fz, F2, F3, F4) channels.

Comparing the visual and the audiovisual paradigm, we found that the power over the temporal (FT7, T7) and frontal (AFz, AF4) channels was significantly higher in the audiovisual paradigm. Over the parietooccipital channels, however we found that the power was significantly higher in the visual paradigm.

Alpha band

In the visual paradigm we observed significant power decreases over the temporal (T7, T8, TP7, TP8) parietal (CP1, CP2, CP3, CP4), and parietooccipital (PO7, PO8 channels from -500 ms, and from -200 ms over the occipital (Oz, O2) channels

During the audiovisual paradigm, we observed significant power decreases compared to baseline activity over the frontotemporal (FT7, FC5, C6, FC6) and parietal (CPz, CP1, CP2, CP3, CP4) channels from -500 ms, and this significant power decrease intensified from -200 ms.

Comparing the visual and the audiovisual paradigm, we found that the power was significantly higher over the temporal (TP7, T7, FT8, T8) channels during the audiovisual paradigm, but from -100 ms the power was significantly higher during the visual paradigm over the frontal (FCz, FC1) and temporal (CP5, C5) channels.

Beta band

In the visual paradigm, we observed significant power decrease over the parietal (CPz, CP1, CP2, CP3, CP4), and temporal-frontotemporal (CP5, C5, F5, T8) channels, and starting from -100 ms over the parietooccipital (PO8) channels.

During the audiovisual paradigm, we observed significant power decreases over the parietal (CPz, CP1, CP2, CP3, CP4), and frontotemporal (CP5, C5, F5, FC5) channels.

Comparing the visual and audiovisual paradigm, we found that the power was significantly higher in the audiovisual paradigm over the occipital (Oz, O1, O2) and parietooccipital (P7, PO7) channels.

Gamma band

During the visual paradigm, we observed significant power decreases over the temporal (TP7, FT7, FC5, CP5), parietal (CP1, CP3, Pz, C2) and frontal (F2, F3, F5) channels.

During the audiovisual paradigm, we observed significant power decrease compared to baseline activity over the frontotemporal (FC4, FC5, F3, F4) and parietal (CPz, CP1, CP2, CP3) channels, and significant power elevation over the occipital (O1, Oz, O2) and parietoocciptal (PO7, PO8) channels.

Comparing the visual and audiovisual paradigm, we found that the power was significantly higher in the audiovisual paradigm over the frontal (FPz, FP2), occipital (O1, Oz, O2), and parietoocciptal (P7, PO7, PO8) channels.

**Generalization phase of the associative learning paradigm**

Theta band

During the visual paradigm, we observed significant power elevation compared to baseline-activity over the frontal-midline (Fz, FCz, Cz) and parietooccipital (POz, PO7) channels.

In the audiovisual paradigm, we observed significant power elevation over the frontal midline (Fz, FCz, Cz) channels, and significant power decreases over the frontal (AF8, F8) and occipital (Oz, O1) channels.

Comparing the visual and audiovisual paradigm, we found that the power was significantly higher in the visual paradigm over the parietoocipital (POz, PO7) and frontal (FCz, AF8, F8) channels.

Alpha band

In the visual paradigm, we observed significant power decreases over the parietal (CP3, C1, C3, C4), and parietooccpital channels (POz, PO7).

During the audiovisual paradigm, we observed significant power decreases over the temporal-frontotemporal (FT8, F6, FC5), parietal (CP1, CP3, CP4, C4), and starting from -100 ms over the occipital (O2) channel.

Comparing the visual and audiovisual paradigm, we found that the power was significantly higher over the occipital-parietooccipital channels (POz, PO7, PO8, O2, O1) in the audiovisual paradigm.

Beta band

During the visual paradigm, we observed significant power decreases over the parietotemporal (T7, FT5, FC5, FC6, C5, CP5), and parietooccipital (P7, PO4, PO7) channels.

In the audiovisual paradigm, we observed significant power decrease over the parietotemporal (CP3, C3, C4, C5, CP5, FC6) and significant power increases over the frontal (FP2) and temporal (TP8, T7) channels.

Comparing the visual and audiovisual paradigm, we found that the power was significantly higher over the parietooccipital (P8, PO8, PO7) and temporal (T7, FT8) channels in the audiovisual paradigm.

Gamma band

During the visual paradigm, we observed significant power decrease compared to baseline activity over the frontotemporal (FC5, FC6, F6, F8) channels.

In the audiovisual paradigm, we observed a significant power decrease over the parietotemporal (C3, C5, CP3, CP5) channels, and a significant power increase compared to baseline activity over the parietooccipital (POz, PO7, P8) channels.

Comparing the visual and audiovisual paradigm, we found that the power was significantly higher over the frontotemporal (F5, F7) and parietooccipital (PO3, PO7, PO8) channels in the audiovisual paradigm.
